# Implication of JAK1/STAT3/SOCS3 Pathway in Aging of Cerebellum of Male Rat: Histological and Molecular study

**DOI:** 10.1038/s41598-020-64050-z

**Published:** 2020-06-01

**Authors:** Enas Ahmed Mohamed, Walaa Mohamed Sayed

**Affiliations:** 1 Department of Anatomy, College of Medicine, Qassim University, Meleda, Buraydah, Saudi Arabia; 2 Department of Anatomy and Embryology, Faculty of Medicine, Kasr Al-Ainy, Cairo University, Cairo, Egypt

**Keywords:** Cells, Genetics research

## Abstract

Aging causes morphological and functional changes in the cerebellum. This work aimed to demonstrate the implication of JAK1/STAT3/SOCS3 on aging–induced changes of rat cerebellum. Thirty male rats were divided into: adult (12 months), early senile (24 months) and late senile (32 months) groups. Immunohistochemical reaction of the cerebellum to GFAP and caspase-3 was assessed and the expression of JAK1, STAT3, SOCS3 proteins was also evaluated. TNFα as well as the activities of malondialdehyde (MDA) and reduced glutathione (GSH) in cerebellar tissue were also measured. The cerebellum of late senile rats revealed more degenerative changes than early senile rats in the form of increase in GFAP and caspase-3 immunoreaction. Additionally, there was decrease in JAK1and STAT3 expression in early and late senile rats and increase in SOCS3 when compare early and late senile groups with adult one. Enhancement of TNFα was noticed with aging as well as significant decrease in GSH and increase in MDA in early senile group. Moreover, late senile group revealed significant decrease in GSH and increase in MDA. It could be concluded that aging resulting in variable changes of the cerebellum as detected by morphological changes, immunohistochemical reactions of caspase-3 and GFAP and expression of JAK1/STAT3/SOCS3 proteins. Additionally, inflammatory marker TNFα and the activity of oxidative/antioxidative stress markers; malondialdehyde (MDA) and reduced glutathione (GSH) were also affected with aging.

## Introduction

The cerebellum is a fundamental part of the brain that is responsible for movement and awareness activities, such as training of motor activity, recognition of time and fine movements^1^. The cerebellar cortex acts for designing motor functions, storing memories and adapting some behaviors, so it is the focus of the different research aspects including structural morphology, components of nerve cell and connections of fibers^2^.

Astroglial cells play a crucial role in aging-induced changes of the cerebellar cortex of rat; astrocyte is a kind of glial cells that is largely affected by aging more than other glial cells. GFAP is extensively used as an indicator of astroglia as it is mostly present in fibrous astrocytes as one of their filamentous content^3^. Astrogliosis or gliosis is an astroglial proliferation that is accompanied by several brain injuries including aging and the regulation of gliosis is related to changes in GFAP expression^4^.

Caspase-3 is one of cysteine proteinases family that mediates apoptosis in a successive manner by destructing the related components within the cell. Additionally, caspase-3 is mediated by death receptor on the cell surface and mitochondrial apoptosis pathways^5,6^.

Signal transducers-Janus kinase (JAK) and transcription activators (STAT) are newly discovered proteins that their intracellular signaling pathway affects the replication of DNA and presentation of genetic material that are included in cellular stimulation, multiplication, immunological reactions and programmed cell death. The JAK-STAT link controls the transport of knowledge and chemical signals from the cytoplasm to the nucleus through the plasma membrane mediated by the STAT proteins^7^.

The signaling pathway of JAK-STAT in the brain is primarily associated with regulation of gene during development, release of hormone, inflammatory processes or carcinogenesis. The different types of JAK and STAT are presented in many regions of the central nervous system, like cerebrum, hippocampal tissue and cerebellar cortex^8^.

The pathway of JAK-STAT could be controlled by many factors as SOCS protein; also known as suppressor of cytokine signaling that can negatively affect the duration of action and severity of cytokine-induced JAK/STAT signaling^7^. Oxidative stress has a crucial effect in the pathology of normal aging. It was found that STAT3 protects from a variety of stresses that induce the formation of reactive oxygen species (ROS)^9^.

Aging is associated with decreased immunity and chronic inflammation^10^. Senescent cells act as a source of chronic inflammation due to senescence-associated secretory phenotype (SASP) secreted by those cells such as inflammatory cytokines and chemokines, which are mediators of inflammation and intracellular signaling^11^.

Aging causes morphological and functional changes in the cerebellum^12^. The cerebellar ultrastructure, immunohistochemical and molecular roles and signaling that explain the pathological effects of aging on cerebellar cortex are not yet completely studied.

The present study aimed to demonstrate the impact of JAK1/STAT3/SOCS3 signaling link on the age-related histological and ultrastructural changes of the cerebellar cortex of male rat. In addition, the study analyzed the age-related cerebellar immunohistochemical expression of GFAP and caspase-3, inflammatory process by measuring TNFα as well as the activity of oxidative/antioxidative stress markers; malondialdehyde (MDA) and reduced glutathione (GSH).

## Material and methods

### **Animals and experimental design**

This study was carried out on thirty *Wistar* male albino rats; the sample size was calculated by G*Power 3.1.9.4. The rats were obtained from the Animal and Experimental house of Faculty of Medicine, Cairo University, Egypt. The animals were environmentally acclimatized for 5 days before euthanization and the international rules for handling, care and use of rats in the experimental study were followed with allowance of free access to drink water and rat pellet diet. The rats were housed in metal cages measured 41 × 29 × 16 cm under standard diurnal conditions of light/dark cycle at 14 h: 10 h with temperature at 22 ± 2 °C. The approval for the experimental design was taken from Cairo University Institutional Animal Care and Use Committee, Egypt (CU/III/F/68/19). This included the method of animal handling, and euthanasia. All the precautions were followed to minimize animal suffering. The guidelines for the experimental processing were in accordance with the standards of National Guide for Care and Use of Laboratory Animals (NIH Publications No. 8023, revised 1978).

The rats were divided into three age groups as follows:

**Group I (adult):** this group consisted of 10 rats of 12 months age.

**Group II (early senile):** this group consisted of 10 rats of 24 months age.

**Group III (late senile):** Consisted of 10 rats of 32 months of age.

The rats of 12, 24, 32 months age were parallel to thirty, sixty, eighty years old in humans, respectively^13^.

### **Tissue sampling**

#### Light microscopic examination

The rats were euthanized at 12 pm by single intraperitoneal injection of sodium pentobarbital at dosage of 60 mg/kg bw. The skulls were opened and the cerebella from 5 rats of each group were put immediately in a fixative solution of ten percent of formol saline and kept for one day at the standard atmospheric temperature of the laboratory center. After that, the cerebellar tissues were rinsed in ascending concentrations of alcohol (70%, 90%, 100%) for dehydration followed by immersion in xylene lasting 2 hrs. Tissues were embedded in paraffin blocks; at first left in loose wax followed by hard wax at 60°C for further 1 hr, then, 4-5 µm thickness sections were cut for the following.

##### Hematoxylin and eosin staining

To study the histological structure of cerebellum in all groups by using light microscopic examination.

##### Immunohistochemical study

Immunostaining was done by dehydration and de-waxing of paraffin blocks then cerebellar sections of 5 µm thick were cut and buffered with 10-mM sodium citrate for antigen retrieval. 0.9% hydrogen peroxide in absolute methanol was used for blocking activity of internal peroxidase. Not related antibodies were blocked by addition of protein for 1 h (ab64226, Abcam, Cambridge, UK). Incubation of sections was done with mouse monoclonal GFAP antibodies (Abcam, Cambridge, UK) 1:20 dilution and rabbit monoclonal caspase-3 antibodies (Abcam, Cambridge, UK), 1:200 dilutions for 1 h. Sections were rinsed in phosphate buffered saline (PBS) then heated with Rat IgG antibody (ab150160, Abcam, Cambridge, UK), followed by washing in PBS and incubation with Novolink polymer (Novo-castra). Deletion of the primary antibody in automated staining design and tonsillar human tissue were done for the 2 negative controls for GFAP and caspase-3.

##### Histomorphometric measurement of GFAP and caspase-3 immunoreacted sections

The area percent of GFAP-immunostained astrocytes as well as caspase-3 cytoplasmic immunoreaction was measured in 10 non-overlapping microscopic fields picked randomly from each slide. Four sections were analyzed for one cerebellum and this technique was applied to the cerebellum of each rat in a given group, *i.e*. 40 measurements were taken from each rat group. This histomorphometric measurement was assessed by using image analyzer (Leica Qwin 500 software Image computer system Ltd., Cambridge, England). Images were captured live on to the screen from sections under a light microscope (Olympus Bx-40, Olympus Optical Co. Ltd., Japan) then the video images were digitalized. The cells stained with dark brown colour were considered positive.

#### Electron microscopic examination

From the other 5 cerebellar cortices from each group, 1mm^3^ in thickness was immediately fixed in 3% glutaraldhyde, buffered with phosphate buffered saline (PBS) and processed for preparation of semithin slices. Ultrathin sections were prepared and mixed with uranyl acetate and lead citrate. Transmission electron microscopic (TEM) analysis was carried out by a JEM-1400 (JEOL, Tokyo, Japan).

Detection of JAK1, STAT3 & SOCS3 by Western Blotting method (usingV3 Western Workflow™ Complete System, Bio-Rad® Hercules, CA, USA). Extraction of proteins from cerebellar tissue homogenates was done by using ice-cold radioimmunoprecipitation assay (RIPA) buffer then centrifuged at 12,000 rpm for twenty minutes. Similar concentrations of protein (20-30µg of total protein) were separated by 10% acrylamide gel electrophoresis and transferred to polyvinylidene difluoride membranes (Pierce, Rockford, IL, USA). The membranes were rinsed with PBS after transfer and were blocked with 5% skimmed milk powder in PBS for one hour at room temperature. The blots were formed using antibodies for JAK1, STAT3, SOCS3 and beta actin gained by (Thermoscientific, Rockford, Illinois, USA) and heated at 4 °C overnight with slight shaking. After washing, secondary antibodies with marked peroxidase were added, and the membranes were heated for one hour. Band intensity was analyzed by ChemiDocTM imaging system with Image LabTM software version 5.1 (Bio-Rad Laboratories Inc., Hercules, CA, USA).The results were expressed following normalization for β-actin protein expression.

Measurement of TNF alpha by ELISA. The kit was supplied by MyBiosource, USA.

##### Measurement of oxidative marker (malondialdehyde-MDA)

One hundred milligrams of the cerebellar tissue were homogenized in 1 mL phosphate buffered saline, pH 7.0 with the micropestle. 20% Trichloroacetic acid (TCA) was added to cerebellar tissue homogenate precipitating the protein and then centrifuged. Thiobarbituric acid (TBA) solution was added to the supernatants after their collection then was boiled for 10 minutes in a water bath then the absorbance was measured. The concentration of MDA in supernatants of cerebellar homogenate was calculated using the standard curve^14^.

##### Measurement of antioxidant marker (reduced Glutathione-GSH)

It depends on the reduction of 5,5dithiobis (2-nitrobenzoic acid) (DTNB) with reduced glutathione (GSH) producing a yellow compound. The reduced chromogen is directly proportional to GSH concentration and its absorbance can be measured at 405 nm by using its kit^15^.

##### Estimation of the thickness of the layers of cerebellar cortex

The thickness of the different layers of the cerebellar cortex (the molecular and granular layers) was measured using Leica Qwin 500 LTD image analyzer computer system (software Qwin 500, Cambridge, UK). In each animal of the different age groups, the used sections were stained with hematoxylin and eosin under magnification X 400.

### **Data analysis**

The data obtained from the image analyzer were expressed as mean ± SD and analyzed using the SPSS (Statistical Package for Social Sciences), version 21, Chicago, USA. The data were subjected to one–way ANOVA test for comparison between the different groups. Results were considered statistically significant with the value of (*p* ≤ 0.05) and highly significant with the *p* value ≤ 0.001. Data was tabulated and represented graphically. The difference; either increase or decrease in all studied parameters were expressed as a percent by using special statistical equation.

### Ethical approval

All applicable international, national, and/or institutional guidelines for the care and use of animals were followed.

## Results

### **Aging–induced histological changes in the cerebellum**

**Figure 1:**
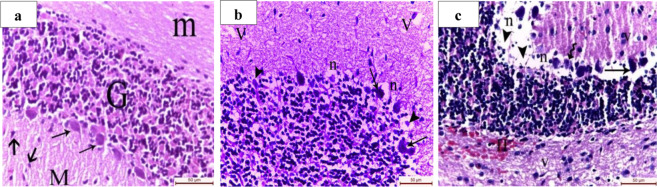
Light micrographs of cerebellar sections stained with hematoxylin and eosin X 400. (**a**) Adult male albino rat showing the three layers; granular layer (G) formed of tightly packed small rounded cells, molecular layer (M) formed of purkinjie cells dentritic fibers, scattered basket and stellate cells (thick arrows) and purkinjie cell layer arranged as one row between the granular layer and the molecular layer. The purkinjie cells (thin arrows) appear as large piriform cell bodies with central nuclei. (**b**) Early senile rats revealing areas of complete disappearance of purkinjie cells. Some purkinjie cells appear almost completely destroyed (arrowheads), irregular in shape with deeply stained cytoplasm (arrows) and surrounded with vacuolated neuropil (n). Note the vacuolated areas (V) in the molecular layer. (**c**) Late senile rats showing areas of complete loss of the purkinjie cell layer (arrowheads). The few remaining purkinjie cells appeared shrunken (wavy arrow), irregular in shape with deeply stained cytoplasm (arrow) and surrounded with vacuolated neuropil (n) Note the vacuolated areas (V) in the molecular and the granular layers (V) and the interstitial hemorrhage (H) in the medulla. (Scale bar is *50 µm* in all images, *n* value = 10 rats for each age group).

Microscopic examination of H&E-stained cerebellar tissue showed that early senile rats (group II) exhibited degenerative changes including damaged purkinjie cells which were surrounded with vacuolated neuropil, areas of complete disappearance of purkinjie cells, and vacuolated areas in the molecular layer. The hematoxylin and eosin – stained sections of the late senile rats of group III revealed areas of complete loss of the purkinjie cell layer. The few remaining purkinjie cells appeared destroyed and surrounded with vacuolated neuropil. Vacuolated areas in the molecular and the granular layers were noticed. Additionally, interstitial hemorrhage in the medulla was detected (Fig. 1a–c).

### **Effect of aging on density of glial fibrillary acidic protein (GFAP) immunoreaction in the granular layer of the cerebellar cortex**

**Figure 2:**
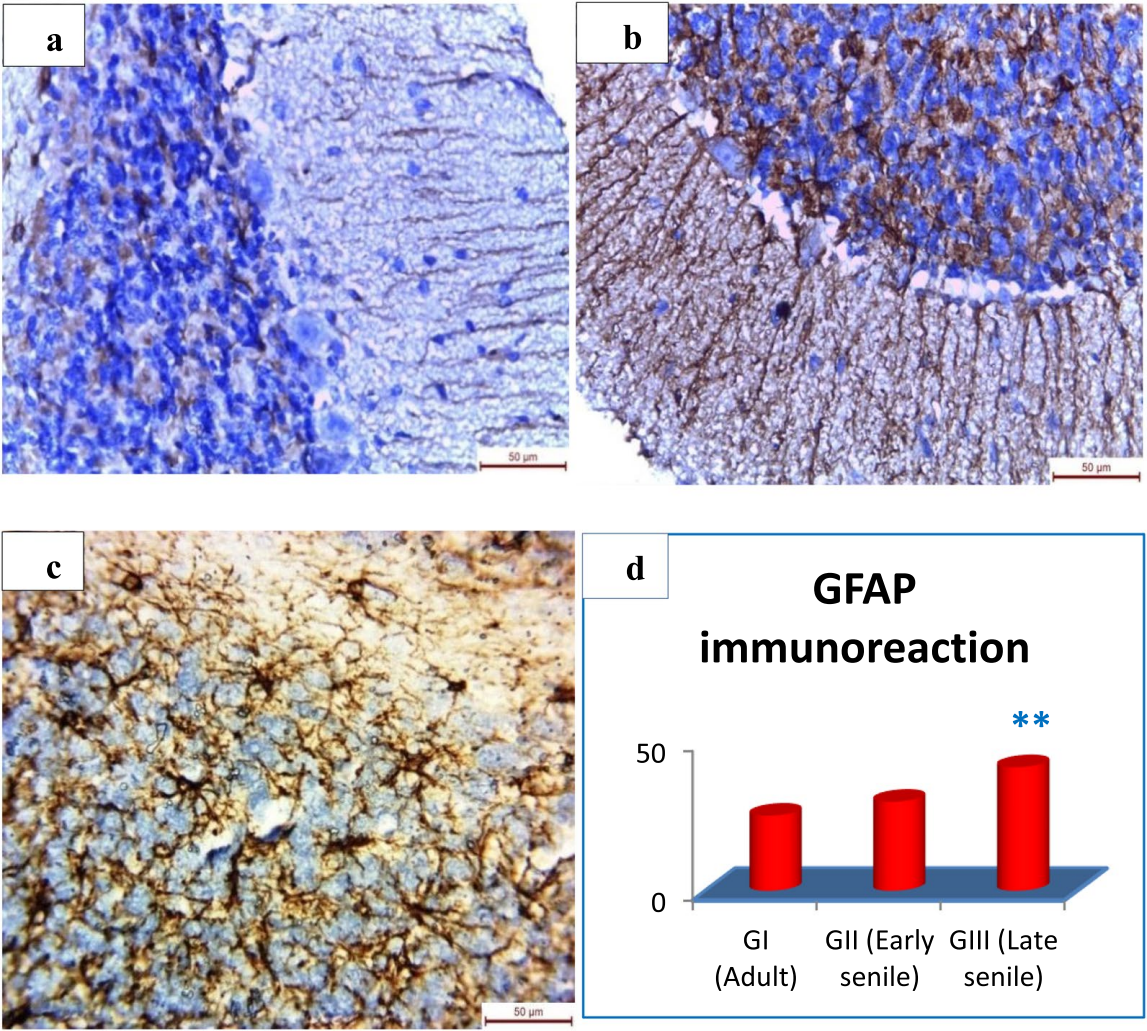
Light micrographs of granular layer of cerebellum with immunohistochemistry by anti-GFAP antibodies. (**a**) In adult male albino rat, the cell bodies of astrocytes exhibiting slight GFAP immunoreaction. (**b**) Early senile rats showing mild increase in GFAP immunoreactivity (early senile versus adult *p* = 0.21). (**c**) Late senile group revealing extensive increase in number and intensity of GFAP immunoreaction (X 400). (**d**) Mean ± SD of GFAP immunoreaction in all groups; (**) a highly statistically significant increase in late senile versus either adult or early senile (*p *≤ 0.001). (Scale bar is *50 µm* in all images, *n* value = 10 rats for each age group).

Immunohistochemical study of GFAP in the cerebellar cortex sections revealed astrocytes that appeared darkbrown in the granular layer. There was slight GFAP immunoraction in adult group (Fig. 2a), while in early senile group; there was mild increase in GFAP (Fig. 2b). Late senile group revealed extensive increase in density of GFAP in the granular layer (Fig. 2c). There was high significant increase (*p* ≤ 0.001) in GFAP immunoraction in late senile rats as compared to either adult rats or early senile rats by 65% and 39%, respectively. However, there was statistically non-significant difference (*p* = 0.21) regarding GFAP immunostaining in early senile rats as compared to adult one (Fig. 2d, Table 1).

**Table 1:** Effect of aging on GFAP and caspase-3 immunoreaction in all groups.

	Adult Mean ± SD	Early senile Mean ± SD	Late senile Mean ± SD	*P* value
GFAP	25.0500 ± 2.55	29.7133 ± 1.90	41.3167 ± 6.46	Early senile versus adult *p* = 0.21 NS Late senile versus adult *p* < 0.001** Late senile versus early senile *p* = 0.001**
Caspase-3	23.7533 ± 1.44	29.71 ± 2.08	42.50 ± 5.11	Early senile versus adult *p* = 0.021^#^ Late senile versus adult *p* < 0.001 ** Late senile versus early senile *p* < 0.001**

*SD:* standered deviation; *NS*: non-significant (*p* > 0.05); ^**#**^statistically significant (*p* ≤ 0.05); **highly significant (*p* ≤ 0.001).

### **Assessment of aging–induced cerebellar apoptosis by immunohistochemical study of caspase-3**

**Figure 3:**
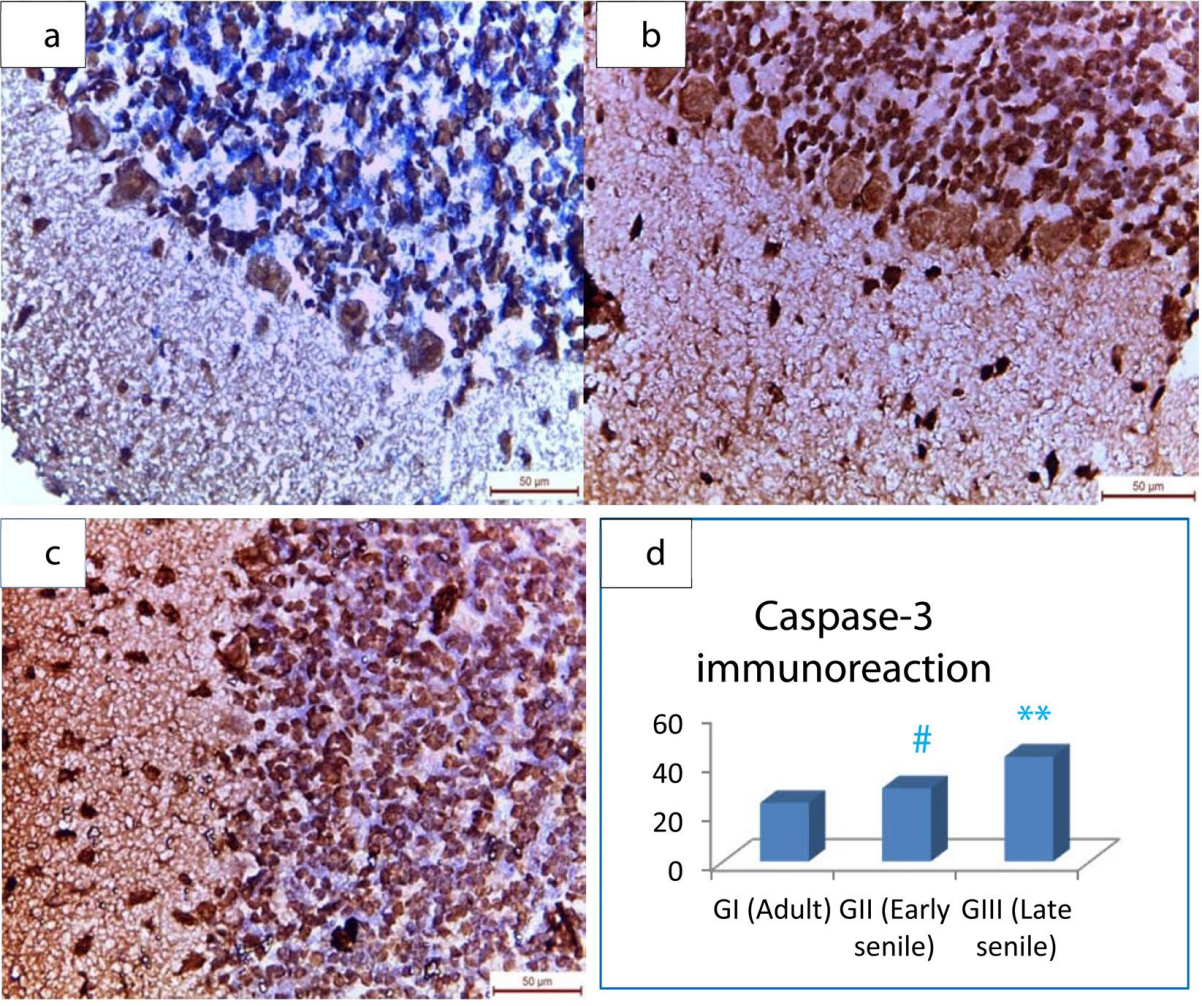
Light micrographs of cerebellar sections with immunohistochemistry by anti-caspase 3 antibodies. (**a**) Adult male albino rat demonstrating mild caspase-3 immunoreactivity in the three cerebellar layers. (**b**) Early senile rats showing moderate increase in caspase-3 immunostaining cells in the three layers of the cerebellum. (**c**) Late senile rats exhibiting extensive caspase-3 immunoreaction in the three cerebellar layers (X 400). (**d**) Mean ± SD of caspase-3 immunoreaction in all groups; (**) a highly statistically significant increase in late senile versus either adult or early senile (*p* < 0.001), (**#**) a statistically significant increase in early senile versus adult (*p* = 0.021) (Scale bar is *50 µm* in all images, *n* value = 10 rats for each age group).

The sections from the cerebellum reacted immunohistochemically to caspase-3 as follows: group I (adult male rat) revealed mild caspase-3 cytoplasmic immunoreaction (Fig. 3a). However, the sections from group II (early senile rat) demonstrated an increase in caspase-3 immunoreactivity localized in the cytoplasm of the cerebellar cells (Fig. 3b). In addition, the examination of the specimens from groups III (late senile) showed extensively caspase-3 cytoplasmic immunoreactivity (Fig. 3c). There was statistically significant moderate increase (*p* = 0.021) by 25% in caspase-3 immunoraction in early senile rats as compared to adult rats, also there was highly statistically significant increase (*p* < 0.001) regarding caspase-3 immunostaining in late senile rats as compared to either adult or early senile rats by 79% and 43%, respectively (Fig. 3d, Table 1).

### **Aging–induced ultrastructural changes in the cerebellum**

**Figure 4:**
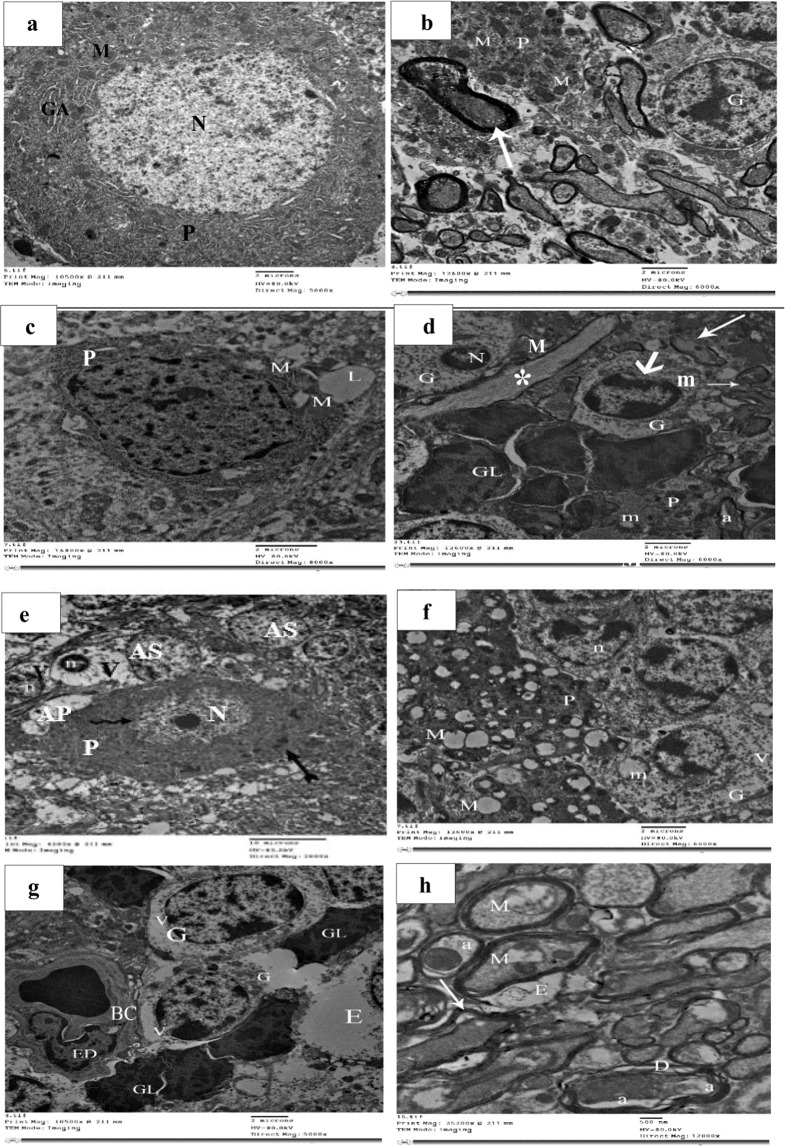
(**a, b**) Electron micrographs from rat cerebellum of group I (adult) showing purkinjie cell (P) with euchromatic nucleus (N).The perikaryon displaying healthy mitochondria (M) and Golgi apparatus (GA). Granular cell (G) and myelinated nerve axons with regular compact myelin sheath (arrow) are also seen. (TEM; a X 5000, b X 6000). (c, d) Electron micrographs from rat cerebellum of group II (early senile) revealing purkinjie cell (P) containing mitochondria with disrupted cristae (M). Note the lipid droplet (L) in the surrounding neuropil. Granular cells (G) with shrunken nucleus (N), mitochondria with disrupted cristae (m), indented nuclear membrane (thick arrow) and many glial cells (GL) are also seen. Note the disrupted myelin sheath (thin arrows) and the vacuolated axoplasm (a) and longitudinally-cut ramified dendrite of purkinjie cell (asterisk). (TEM; c X 8000, d X 6000, *n* value = 10 rats for each age group). **(e–h)** Electron micrographs from rat cerebellum of group III (late senile). (e) Demonstrating purkinjie cell (P) containing shrunken nucleus (N) with irregular membrane (wavy arrow), irregular nucleolus and numerous cytoplasmic lysosomes (double splitted arrow). Many astrocytes (AS) with shrunken nucleus (n) and markedly vacuolated cytoplasm (V) are also seen. Note the swollen astrocytic process (AP). **f**) Revealing many ballooned mitochondria (M) within the purkinjie cell as well as granular cells (G) with irregular nucleus (n), vacuolated cytoplasm (V) and ballooned mitochondria with disrupted cristae (m) are also observed. **g**) Displaying granular cells (G) with vacuolated cytoplasm (V) and congested blood capillary (BC) . The endothelial cell (ED) showing irregular nucleus. Intercellular edema (E) and many glial cells (GL) are noticed. **h)** Showing disrupted (D) and splitted (arrow) axonal myelin sheath, vacuolated axoplasm (a), mitochondria with disrupted cristae (M) and edema surrounding the axons (E). (TEM; e X 2000, f X 6000, g X 5000, h X 12000, *n* value = 10 rats for this age group).

Electron microscopic examination of adult rat cerebellar cortex revealed purkinjie cell with euchromatic nucleus. The perikaryon demonstrated healthy mitochondria and Golgi apparatus. There were normal granular cell and regular myelin sheath of the myelinated nerve axons (Fig. 4a,b).

Cerebellar sections from early senile rats using electron microscope revealed purkinjie cell had mitochondria with disturbed cristae. The granular cells demonstrated shrunken nuclei, indented nuclear envelope and disturbed mitochondrial cristae as well as many glial cells. The axons showed disrupted myelin sheath and vacuolated axoplasms. Lipid droplets surrounding neuropil were noticed (Fig. 4c,d).

Ultrastructural examinations of cerebellar sections of the late senile rats demonstrated purkinjie cell with shrunken nuclei and irregular nuclear envelope, ballooned mitochondria and numerous cytoplasmic lysosomes. The granular cells showed markedly vacuolated cytoplasm with shrunken irregular nuclei and damaged mitochondria. Many glial cells as well as congested blood capillaries with damaged endothelial cell nuclei and interstitial edema were also found. The axons demonstrated disrupted and splitted myelin sheaths, vacuolated axoplasms, mitochondria with disrupted cristae and edema surrounding the axons (Fig. 4e–h).

### **Modulation of the thickness of the different layers of cerebellum with aging**

**Figure 5:**
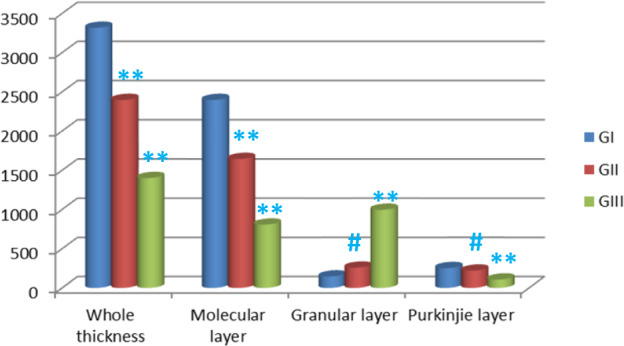
Bar chart displaying mean values of the thickness of different layers of cerebellar cortex in all groups; the whole cerebellar thickness and molecular layer showing (**) a highly statistically significant decrease in both late and early senile groups versus either each other or adult group (*p *< 0.001). The granular layer displaying (**) a highly statistically significant increase in late senile group versus either adult or early senile groups (*p* < 0.001), (#) moderately statistically significant increase in early senile group versus adult group (*p* = 0.012). The purkinjie layer revealing (**) a highly statistically significant decrease in late senile group versus either adult or early senile groups (*p* < 0.001), (#) moderately statistically significant decrease in early senile group versus adult group (*p* = 0.027). *(n* value = 10 rats for each age group).

Morphometric measurement of the different layers of the cerebellum revealed statistically highly significant decrease (*p* < 0.001) in the whole thickness of the cerebellar cortex with early senile and late senile groups by 28% and 58%, respectively as compared to adult group. Moreover, high significant decrease (*p* < 0.001) by 41% was observed in late senile group as compared with early senile one. Additionally, there was highly significant decrease (*p* < 0.001) in the molecular layer of the cerebellar cortex of rat with aging (early senile and late senile) by 31% and 66%, respectively as compared to adult group. Furthermore, significant decrease by 51% was noticed high (*p* < 0.001) in the molecular layer of late senile group when compared with early senile group. There was statistically significant increase (*p* = 0.012) by 78% in the granular layer in early senile group as compared to adult one and highly significant increase (*p* < 0.001) in this layer in late senile group as compared to either adult or early senile groups by 4 and 2 folds, respectively. In addition, moderate significant decrease (*p *= 0.027) by 13% was noticed in the purkinjie layer of early senile group in comparison with adult one and high decrease (*p* < 0.001) was observed in the purkinjie layer in late senile group as compared to either adult or early senile groups by 58% and 52%, respectively (Fig. 5, Table 2).

**Table 2:** Effect of aging on the thickness (***µ*m**) **of the different layers of the cerebellar cortex in the different groups**.

Thickness	Adult Mean (*µm*) ± SD	Early senile Mean (*µm*) ± SD	Late senile Mean (*µm*) ± SD	*P* value
Whole thickness	3316.77 ± 187.53	2395.8 ± 35.79	1400.43 ± 210.2	Early senile versus adult *p* < 0.001** Late senile versus adult *p* < 0.001 ** Late senile versus early senile *p* < 0.001**
Molecular layer	2395.60 ± 35.79	1643.74 ± 82.22	807.75 ± 46.009	Early senile versus adult *p* < 0.001** Late senile versus adult *p* < 0.001 ** Late senile versus early senile *p* < 0.001**
Granular layer	144.04 ± 54.90	257.20 ± 24.24	994.78 ± 82.27	Early senile versus adult *p* = 0.012^#^ Late senile versus adult *p* < 0.001 ** Late senile versus early senile *p* < 0.001**
Purkinjie layer	251.36 ± 32.14	219.36 ± 5.71	105.18 ± 0.3.99	Early senile versus adult *p* = 0.027^#^ Late senile versus adult *p* < 0.001** Late senile versus early senile *p* < 0.001**

*SD*: standered deviation; *NS*: Non significant (*p* > 0.05); ^#^statistically significant (*p* ≤ 0.05); **highly significant (*p* ≤ 0.001).

**Figure 6:**
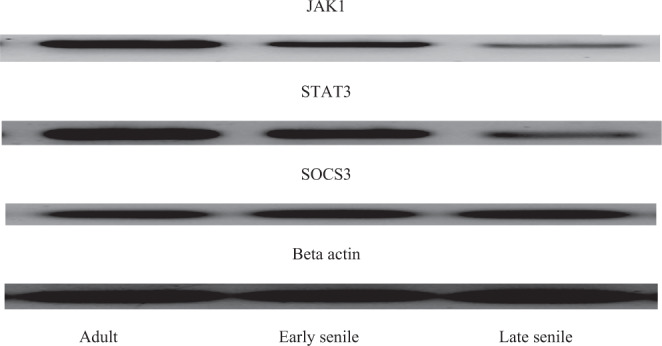
Expression of JAK1, STAT3 and SOCS3 proteins in adult, early senile and late senile groups assessed by western blotting and Beta actin is used as loading control.

**Figure 7:**
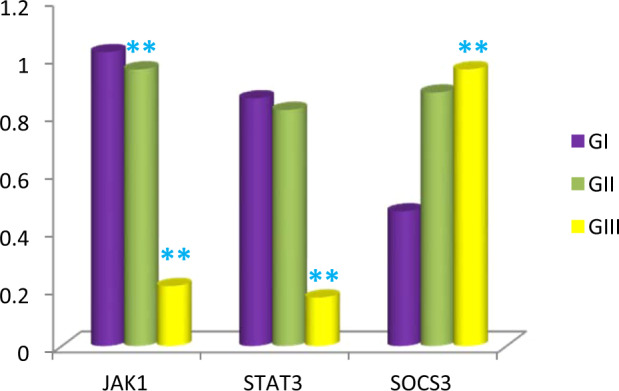
Bar chart showing the mean of JAK1, STAT3 and SOCS3 in adult, early senile and late senile groups; JAK1 showing (**) a highly statistically significant decrease in both late and early senile groups versus either each other or adult group (*p *≤ 0.001). Mean values of STAT3 revealing (**) highly statistically significant decrease in late senile group versus either adult or early senile groups (*p* < 0.001). Mean values of SOCS3 displaying (**) a highly statistically significant increase in late senile group versus either adult or early senile groups (*p *< 0.001). *(n* value = 10 rats for each age group).

Changes of JAK1, STAT3 and SOCS3 signaling pathway with aging: (Figs. 6, 7 and Table 3).

**Table 3:** Effect of aging on the JAK1, STAT3, SOCS3, TNFα, GSH and MDA in all groups.

	Adult Mean ± SD	Early senile Mean ± SD	Late senile Mean ± SD	*P value*
JAK1	1.02 ± 0.014	0.96 ± 0.033	0.21 ± 0.011	Early senile versus adult *p* = 0.001** Late senile versus adult *p *< 0.001 ** Late senile versus early senile* p *< 0.001**
STAT3	0.86 ± 0.018	0.84 ± 0.032	0.17 ± 0.014	Early senile versus adult* p* > 0.05NS Late senile versus adult *p* < 0.001 ** Late senile versus early senile *p *< 0.001**
SOCS3	0.47 ± 0.018	0.51 ± 0.054	0.96 ± 0.018	Early senile versus adult *p *> 0.05NS Late senile versus adult* p* < 0.001 ** Late senile versus early senile *p* < 0.001**
TNFα	45.23 ± 0.16	79.08 ± 15	110.28 ± 0.23	Early senile versus adult* p* < 0.001** Late senile versus adult *p* < 0.001 ** Late senile versus early senile *p* < 0.001**
GSH	38.25 ± 0.18	31.85 ± 0.52	17.25 ± 0.18	Early senile versus adult* p* < 0.001** Late senile versus adult *p *< 0.001 ** Late senile versus early senile p < 0.001**
MDA	29.25 ± 0.18	32.71 ± 0.76	76.04 ± 0.091	Early senile versus adult *p *< 0.001** Late senile versus adult* p* < 0.001 ** Late senile versus early senile* p* < 0.001**

*SD:* standered deviation; *NS*: Non significant (*p* > 0.05); ^#^statistically significant (*p* ≤ 0.05); **statistically highly significant (*p* ≤ 0.001).

Western blotting technique was used to identify the activities of JAK1, STAT3 and SOCS3 proteins in the cerebellar homogenates. There was statistically significant decrease (*p* = 0.001) in the expression of JAK1 by 6% in the early senile group as compared with adult group. Additionally, the decrease in JAK1 was high (*p* < 0.001) when compare late senile group with either adult or early senile groups by 79% and 78%, respectively.

Regarding STAT3, there was statistically highly significant decrease (*p* < 0.001) in late senile group as compared with either adult or early senile groups by 80% and 79%, respectively. However, statistically non-significant difference was observed between early senile and adult groups regarding STAT3.

The increase in SOCS3 was statistically high (*p* < 0.001) when compare the late senile group with either adult group by one and half fold or early senile group by 88%. However, there was non-difference between early senile and adult groups.

### **Assay of TNFα as a marker of inflammatory induction by aging**

**Figure 8:**
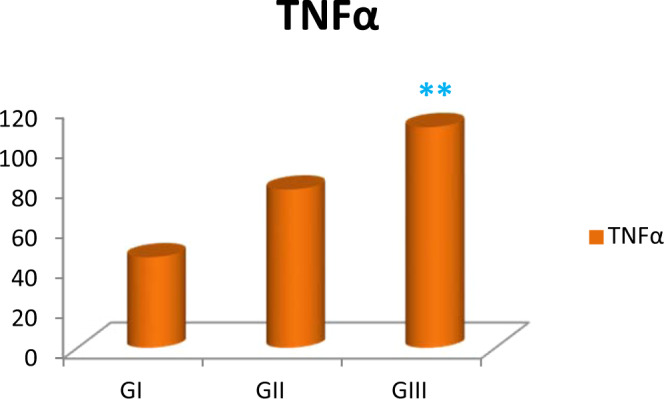
Bar chart revealing the mean of TNFα in adult, early senile and late senile groups; (**) a highly statistically significant increase in late senile versus either  early senile or adult groups. (#) highly statistically significant increase in early senile group versus  adult group (*p* < 0.001). *(n* value = 10 rats for each age group).

ELISA displayed an enhancement of TNFα with aging which was confirmed statistically; early senile and late senile groups revealed highly statistically significant increase (*p* < 0.001) as compared with adult one by 75% and three-fold increase, respectively. In addition, late senile group demonstrated an increase in TNF**α** by 39% when compared with early senile group (Fig. 8, Table 3).

### **Modulation of antioxidant marker (GSH) and oxidant marker (MDA) with aging**

**Figure 9:**
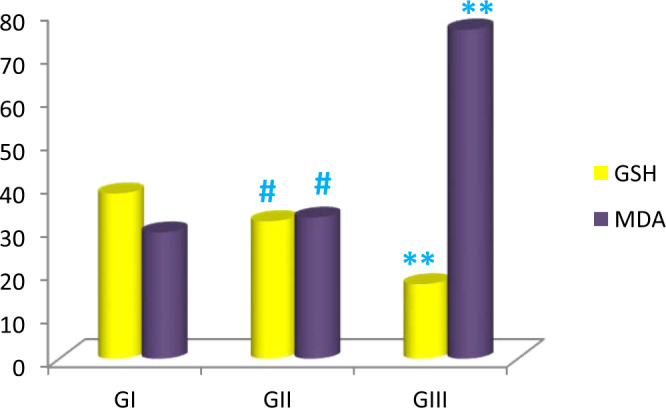
Bar chart demonstrating the mean of GSH and MDA in adult, early senile and late senile groups; (#) a highly statistically significant (*p* ≤ 0.001) decrease in GSH and increase in MDA in early senile group versus adult group. (**), a highly statistically significant decrease in GSH and increase in MDA in late senile group versus either adult or early senile groups. *(n* value = 10 rats for each age group).

There was statistically significant (*p* ≤ 0.001) decrease in GSH and increase in MDA in early senile group as compared with adult group by 16.7% and 12%, respectively. Moreover, late senile group revealed statistically significant decrease in GSH and increase in MDA as compared to either adult one by 55% and 5 folds or early senile group by 46% and 4 folds, respectively (Fig. 9, Table 3).

## Discussion

The present research assessed aging-induced histological, immunohistochemical and ultrastructural changes of cerebellum. The study emphasized on the age modulation of the cerebellar JAK1/STAT3/SOCS3 as measured by western blotting technique. In addition, the detection of oxidant and antioxidant markers as well as inflammatory marker induced by normal aging was also evaluated.

Histological examination of cerebellar tissue revealed moderate degenerative changes in early senile rats in the form of damaged purkinjie cells which confirmed by disruption of their mitochondrial cristae as evaluated by the electron microscope. Vacuolated neuropil with scattered areas of complete disappearance of purkinjie cells, and scattered vacuolated areas in the molecular layer were also observed in light microscopic examination of this group. In the late senile rats of group III, there were extensive degenerative changes of the cerebellum in the form of complete loss of the purkinjie cell layer that was confirmed by shrinkage of their nuclei with irregular nuclear envelope as assessed by electron microscope. The few remaining purkinjie cells appeared destroyed and surrounded with vacuolated neuropil. Additionally, ballooned mitochondria and numerous cytoplasmic lysosomes were noticed in ultrathin sections of late senile group. Extensive vacuolated areas in the molecular and granular layers were observed, additionally, interstitial hemorrhage was detected. The granular cells appeared in the ultrathin sections of the late senile group with markedly vacuolated cytoplasm, shrunken irregular nuclei and damaged mitochondria.

Henrique *et al*.^16^ reported no age-related mitochondrial changes, however regression in the mitochondrial physiology with aging was found by several studies^17,18^. This functional decrease was attributed to continuous cytoplasmic collection of Ca^2+^ that resulting in dangerous effects such as apoptosis^19^. Squier and Bigelow^20^ added that the mitochondria of senile mice reveal activation of permeability transition pore (PTP) that resulting in enhanced formation of mitochondrial oxidant agents with increased intracellular Ca2+ and release of pro-apoptotic components.

Luo *et al*.^21^ emphasized on the reduction of mitochondria in aged synapses and this decrease may be due to abnormal secretion of neurotransmitter which in turn causing suppression of the electrical impulses from neurons. Earlier, Louis *et al*.^22^ attributed the aging declining in the activity of the purkinjie cells to the insufficient amount of noradrenaline in the synapses, particularly in the cerebellum. Furthermore, levels of neurotransmitter hydrolases decrease, which weakens the activity of glutamate dehydrogenase, causing glutamate accumulation which in turn harm the neurons and can cause further neuronal apoptosis^23,24^.

Morphometric measurement of the different layers of the cerebellum revealed statistically significant decrease of the whole thickness of the cerebellar cortex and its molecular layer with aging (early senile and late senile) as compared to adult group. In addition, statistically significant decrease was noticed in the thickness of the purkinjie layer with aging (early senile and late senile groups) in comparison with adult group. Hara *et al*.^25^ demonstrated significant decrease of purkinjie cell count in aged rats as the purkinjie cells are the most vulnerable and sensitive to the changes of aging. Nearer findings were found by Zhang *et al*.^26^ who reported reduction in total thickness of the cerebellar cortex secondary to reduction in the molecular layer thickness as a result of decrease in its number of neurons (basket and stellate cells). The later neurons act as inhibitory synapses with purkinjie cells and their destruction could suppress the inhibitory impulse to the purkinjie cells. The decrease in cerebellar cortex thickness is considered the most important factor in aging deterioration of the normal physiologic mechanisms of the brain. Hadj-Sahraoui *et al*.^27^ attributed the reduction in molecular layer thickness to the destructive effect of aging on the foliation of the purkinjie cells dendrites.

The current research work revealed significant increase in the granular layer thickness which was moderate in early senile group and severe in late senile group. Also, many glial cells were noticed in electron microscopic examination of early and late senile cerebellar sections. Moreover, GFAP immunoreaction proved the detection of astrocytes in the granular layer of the cerebellar cortex as there was strong positive GFAP immunoreaction in early and late aged rats in comparison with adult rats as well as increase in GFAP immunostaining in late senile rats as compared to early senile one. The increase in the granular layer thickness with aging is due to astrocytes proliferation in this layer. Jalenques *et al*.^28^; Sabbatini *et al*.^29^ and Wu *et al*.^30^ detected astrocytes by GFAP immunoreaction in variable mammalian species and demonstrated proliferation of these cells in response to aging. The changes in the astrocytes could be attributed to the neurodegenerative changes of aging; however, there was no clear mechanism explains the age-induced GFAP in astrocytic cells. Zhang *et al*.^26^ suggested that the enhancement in the size and count of astrocytes may inhibit the damaging mechanism of aged nerve cells and degeneration of the cerebellar cortex. The authors attributed the astrocytic proliferation to their compensatory reaction to the reduction of the cerebellar cortex thickness by occupying the empty spaces produced by the loss of neurons. Earlier, Hilber and Caston^31^ and Caston *et al*.^32^ stated that there is no clear link between reductions in the thickness of the different layers of the cerebellar cortex; however, the suppression in the motor activity with aging is confirmed by the decrease in the count of nerve cells.

Deverman and Patterson^33^ demonstrated that cytokines’ stimulation of JAK-STAT signaling pathway resulting in transformation of the cortical progenitors into mature astrocytes that in turn express the marker glial fibrillary acidic protein (GFAP). Levy and Darnell^34^ revealed that the stimulation of JAK resulting in phosphorylation of STAT proteins which in turn attach to particular DNA binding components within the nucleus to enhance transcription of genes included in glial differentiation.

Attwell *et al*.^2^ reported that the distributed fibers of the granular cells' axons send excitatory impulses to the purkinjie cells. So, the change in the cells of the granular layer with aging will decrease the excitatory impulses to the purkinjie cells. Boyden *et al*.^35^ added that the loss of granular cells could affect the motor activity in aged ones as they are confined to conservation of memories along the different durations, regulation of movement mechanics and control the double directional amplitude of movements.

Immunohistochemical study of adult cerebellar sections of this work revealed high statistically significant increase in caspase-3 immunoraction in late senile animals in comparison to either adult or early senile groups. Also, there was moderate statistically significant increase regarding caspase-3 immunostaining in late aged rats in comparison to adult. Also, Sun *et al*.^36^ found an increase in the brain apoptotic cells with aging mainly in the granular layer. Sastre *et al*.^37^ attributed the aging-induced apoptosis to the increase in the formation of reactive oxygen radicals with age. Dayer *et al*.^38^ emphasized on the crucial role of apoptosis in the brain insults that secondary lead to neuronal loss. Sierra *et al*.^39^ pointed out that apoptotic cells are scavenged by microglial cells that are primarily located in the brain tissue.

In the present work, activities of JAK1, STAT3 and SOCS3 proteins in cerebellar homogenates were detected by using blotted method. There was reduction in JAK1 and STAT3 expression and enhancement in SOCS3 expression in late aged group in comparison with either adult or early aged groups. However, there was statistically non-significant difference was detected between early senile group and adult regarding STAT3 and SOCS3. De-Fraja *et al*.^40^ pointed out that the expression of JAK/STAT proteins differed throughout the developmental duration; their level showed an increase during embryonic period, in particular JAK1 and STAT3 and their level showed gradual decrease during growth and into adulthood. Schwaiger *et al*.^41^ reported that the regeneration of neurons in response to lesion will include the pathway JAK-STAT as the activation and expression of STAT3 is high following axonal injury specifically in regenerating neurons. Moreover, Schindler *et al*.^42^ revealed that stimulation of STAT can induce non-apoptotic proteins transcription. The latter information was coinciding with the result of the present work. Schindler *et al*.^42^ demonstrated that the activation of the ratios of STAT1/STAT3 or STAT1/STAT5 ratio would add to the mechanism of programmed cell death, as STAT3 and STAT5 are more antagonists to apoptosis than STAT1.

In present study, there was an increase in the cerebellar inflammation induced by aging either in early senile or late senile rats as measured by TNFα; a marker of inflammation. Regis *et al*.^43^ confirmed this finding and demonstrated that JAK/STAT link is activated by release of stimulatory items in response to inflammation. Earlier, Schwaiger *et al*.^41^ stated that the pathway of JAK-STAT is included in the regeneration of the neurons and limitation of spreading of inflammation in case of injury by forming scars around it. Yu *et al*.^44^ pointed out relation between JAK/STAT pathway with normal as well as abnormal cellular growth. It was found by Guo *et al*.^45^ that STAT3 is closely related to neuronal survival and that the decrease of STAT3 function could be directly related with neuronal death. Herrmann *et al*.^46^ demonstrated that activation of STAT3 would result in formation of the glial scars (astrogliosis) following injury of CNS, an important step for decrease propagation of inflammation. Furthermore, Smith *et al*.^47^ revealed that removal of SOCS3 enhances regeneration of axons following optic nerve injury. This removal in turn, would stimulate gp130-mediated signaling pathway (involving JAK-STAT).

Planas *et al*.^48^ described inflammation of CNS as a complex mechanism that occurs between different types of brain cells such as microglial, astrocytic, neuronal and endothelial cells. Degos *et al*.^49^ added that inflammation follows stimulation of microglial and astrocytic cells that mediated by cytokines, chemokines or growth factors. Moreover, Vezzani *et al*.^50^ mentioned the crucial role of CNS inflammation in saving integrity of the brain against the injurious items but could be worsens several disorders of the brain; involving cerebral ischemia and neurodegenerative pathologies.

Earlier research by Planas *et al*.^48^. emphasized on the effect of reactive oxygen species and interleukins in activation of STAT1 and STAT3 by JAK1-induced effect. This pathway stimulation aimed to enhance the expression of inflammatory genes. In the present work, there was statistically significant increase of GSH (anti-oxidant) and decrease in MDA (oxidant) in early senile and late senile groups in comparison with adult group. Planas *et al*.^48^ stated that activation of STAT isoform resulting in variable effects on inflammatory processes, proliferation or differentiation as they may be included in nerve cell survival.

The attachment of cytokines to the receptors of on the surface of the cell membrane could stimulate kinase components of JAKs resulting in their activation. The stimulated JAKs phosphorylate tyrosine particles on the receptors resulting in formation of binding locations for stimulator of STAT proteins. Homo- or hetero-dimers of STAT3 were formed as a result of release of stimulated STATS from the receptors. These dimers move to the nucleus and attached to transcription elements to regulate expression of proliferative or anti-inflammatory genes^51^.

The present research concluded that aging resulting in variable changes of the cerebellum; apoptosis was confirmed by caspase-3 marker and aging-induced astrogliosis was detected by GFAP. Additionally, inflammatory marker TNFα and the activity of oxidative/antioxidative stress markers; malondialdehyde (MDA) and reduced glutathione (GSH) were also affected with aging. The expression of JAK1/STAT3/SOCS3 proteins was affected by aging and enhancement of JAK1/STAT3 activity with inhibiting of SOCS3 can act as a promising protective line against age-related cerebellar changes.
